# Exploring long COVID in pediatric patients: clinical insights from a long COVID clinic

**DOI:** 10.3389/fped.2025.1640747

**Published:** 2025-10-15

**Authors:** Dina Kamel, My H. Vu, Jeffery Bender, David Warburton, John C. Wood, Sindhu Mohandas

**Affiliations:** ^1^ Division of Infectious Diseases, Children’s Hospital Los Angeles, University of Southern California, Los Angeles, CA, United States; ^2^ Department of Pediatrics, Children’s Hospital Los Angeles, University of Southern California, Los Angeles, CA, United States; ^3^ Biostatistics and Data Management Core, The Saban Research Institute, Children's Hospital Los Angeles, University of Southern California, Los Angeles, CA, United States; ^4^ Department of Radiology, Children's Hospital Los Angeles, University of Southern California, Los Angeles, CA, United States

**Keywords:** COVID-19, long COVID, post COVID, PASC, COVID 19 vaccine

## Abstract

**Background:**

Long COVID describes the persistence or recurrence of symptoms beyond the acute phase of SARS-CoV-2 infection and is increasingly recognized in children and adolescents. Despite its prevalence, understanding of symptom patterns and the influence of vaccination on disease trajectory in pediatric populations remains limited.

**Methods:**

We conducted a retrospective study of patients aged 0–21 years evaluated at the Long COVID Clinic at Children's Hospital Los Angeles between August 2021 and November 2023. Patients were included if they reported persistent or new symptoms ≥4 weeks after SARS-CoV-2 infection.

**Results:**

A total of 123 patients were enrolled. The mean age was 13.1 years, and 51% were male. Symptom onset occurred a mean of 5 weeks after infection. At presentation, 56% of patients reported symptoms lasting 0–24 weeks, 28% for 25–52 weeks, and 16% for >52 weeks. Fatigue (93%) and headache (70%) were the most prevalent symptoms in both younger (<12 years) and older (>12 years) cohorts. Female patients more frequently reported brain fog, dizziness, palpitations, and postural orthostatic tachycardia syndrome. Overall symptom burden decreased significantly over time (*p* < 0.001). Vaccination status at baseline was not associated with difference in symptom duration on initial presentation (*p* = 0.4). However, among those vaccinated after developing long COVID, 41% reported subjective improvement in the following weeks.

**Conclusion:**

Pediatric long COVID is marked by prolonged, multisystem symptoms. Vaccination may offer symptomatic benefit for some patients, though larger prospective studies are necessary to better define its role.

## Introduction

With the rising cumulative incidence of severe acute respiratory syndrome *coronavirus 2 (*SARS-CoV-2) infection, increasing attention has has focused on the persistence of multi-organ symptoms beyond the acute phase, termed “long COVID”. This refers to signs and symptoms that either persist or emerge following the initial COVID-19 illness and cannot be attributed to another diagnosis (1). Various terms exist in the literature, but in this article, we use “long COVID”.

Persistent symptoms have been observed following SARS-CoV-2 infection across all ages and severities, often impairing daily functioning and health-related quality of life (2, 3). In children, long COVID typically follows a mild acute infection and resolves within several months, but many continue to experience symptoms lasting for months or even beyond a year, causing disability and requiring ongoing medical care and follow-up. These symptoms may persist from the initial illness or newly emerge after recovery, often fluctuating with periods of improvement and relapse (3–5).

The definition of long COVID has evolved since its identification (1). In mid-2021, both the Centers for Disease Control and Prevention (CDC) and the National Institutes of Health (NIH) provided early definitions of long COVID/post-acute sequelae of SARS-CoV-2 infection (PASC), using a time frame of four weeks after acute infection. The NIH defined long COVID as post-acute symptoms beyond four weeks (2). The CDC used the umbrella term “Post-COVID Conditions” (PCC) for health consequences persisting or emerging four or more weeks after infection (6).

In October 2021, the WHO proposed a Delphi-based clinical definition: *Post COVID-19 condition occurs in individuals with a history of probable or confirmed SARS-CoV-2 infection, usually 3 months from the onset of COVID-19, with symptoms and that last for at least 2 months and cannot be explained by an alternative diagnosis* (4). On February 2, 2022, the National Institute for Health and Care Excellence (NICE) defined long COVID as symptoms persisting or emerging after acute illness, including ongoing illness (4–12 weeks) and post-COVID-19 syndrome (≥12 weeks) (7).

In 2024, the National Academies of Sciences, Engineering, and Medicine (NASEM) defined long COVID as an infection-associated chronic condition (IACC) occurring after SARS-CoV-2 infection, present for ≥3 months as continuous, relapsing-remitting, or progressive disease affecting one or more organ systems (8).

Long COVID can affect multiple organ systems with varied symptoms. Common manifestations include fatigue, shortness of breath, cough, chest discomfort, diarrhea, headaches, difficulty with balance or walking, sleep disturbances, joint and muscle pain, generalized weakness, cognitive impairments, heart palpitations, paresthesia, skin rashes, and hair loss (9).

Similar to adults, adolescents and children also report “brain fog”, a term encompassing a range of cognitive impairments. Fatigue is among the most consistently reported symptoms across all ages in multiple studies (5, 10, 11).

Most pediatric studies to date have examined individual symptoms of long COVID, often combining data across broad age ranges or concentrating primarily on adolescents. Consequently, there is limited understanding of how symptoms differ between school-aged children (6–11 years) and adolescents (12–17 years) (11).

A large longitudinal cohort within the Researching COVID to Enhance Recovery RECOVER in Pediatrics study highlighted distinct symptom patterns between these groups, emphasizing the need to evaluate and define long COVID independently within each of these age groups. While some symptoms overlapped, gastrointestinal issues and sleep problems were more common in younger children, whereas anosmia and smell changes predominated in adolescents (12). Key developmental changes in children likely also impact the phenotype of long COVID at various ages, making the identification of long COVID much more challenging.

Currently, there is no established diagnostic biomarker for long COVID, and distinguishing it from other conditions remains a clinical challenge (3).

The underlying mechanisms of long COVID and the impact of COVID-19 vaccination are poorly understood. Potential contributors include varying degrees of organ damage, ongoing low-grade inflammation, and immune dysregulation, such as the production of autoantibodies (13).

Patients often show persistently elevated levels of inflammatory markers (14). The theory that SARS-CoV-2 may persist in a hidden form within the body has been suggested and supported by the detection of viral particles found in various organs post- infection. Vaccination may reduce the risk of long COVID by boosting antibody levels, clearing residual viral reservoirs (15)., and lessening acute illness severity, thereby reducing the likelihood of subsequent organ damage or systemic complications (16).

Management of long COVID involves supportive therapies through both non-pharmacological and pharmacological interventions (3). Management focuses on the most burdensome symptoms, with integrated, multidisciplinary strategies employed for complex or severe cases (3).

Long COVID remains under recognized in Pediatrics. This study aims to characterize the clinical spectrum of long COVID in children at a dedicated long COVID clinic and to explore the impact of vaccination on its development and course.

## Methods

### Study design and setting

We performed a retrospective electronic medical record (EMR) review and data analysis of all patients aged 0–21 years evaluated at the COVID Recovery Clinic at Children's Hospital Los Angeles between August 2021 and November 2023. Institutional Review Board (IRB) approval was obtained prior to study initiation. The COVID Recovery Clinic operates within the Division of Infectious Diseases and is staffed by pediatric infectious diseases specialists.

### Study population

Patients were eligible for inclusion if they presented with persistent or newly emerging symptoms at least four weeks following SARS-CoV-2 infection that significantly impacted their quality of life. We used the 4-week time frame in our study, as defined by the NIH and CDC at the time of enrollment (2, 6). Individuals with alternative diagnoses explaining their symptoms were excluded. Symptom documentation varied by age group: in younger children, symptoms were typically reported by caregivers; in older children and adolescents, symptoms were reported by patients themselves and parents; and in some cases, clinician interpretation was used.

During the study period, 123 children met eligibility criteria and all of them were included in the analysis. Alternative diagnoses which would have constituted exclusion criteria, were not observed among those presenting to the clinic, likely because these patients had already been evaluated by their primary pediatricians and other sub specialists prior to referral. Patients seen in our clinic were either self-referred or referred by their primary pediatricians, with the majority of cases resulting from self-referral. As this study was based on retrospective chart review, variability in clinic attendance resulted in incomplete longitudinal data, with not all patients represented at each follow-up interval.

### Data collection

Data extraction was conducted by a single trained reviewer using a pre-designed Excel template with standardized variable definitions. To ensure data accuracy and reliability, a second independent reviewer randomly verified approximately 10% of abstracted records. Any discrepancies identified during verification were resolved through discussion between the two reviewers, with consultation of the original EMR when necessary.

### Statistical analysis

Demographics and characteristics were summarized using mean and standard deviation (SD) for continuous variables, and frequency and percentage for categorical variables. Differences in the prevalence of each symptom at presentation between males and females, overall and by age groups, were assessed by Fisher's Exact tests, as well as logistic regression models. Although a total of 43 symptoms were recorded, only those reported by more than five patients were included in these comparisons. A penalized likelihood-based method, Firth's bias-reduced logistic regression (R package “logistf”), was fit to accommodate the low prevalence rates (17). As a supplementary analysis, symptoms were grouped into 12 domains, and the same comparisons of prevalence between males and females were repeated at the domain level. The number of recorded symptoms for each patient was summarized at onset, 6-, 12-, 18-, and 18+ months. If a patient had more than one visit in the same time period, the average number of recorded symptoms across the visits was used as the outcome. To examine the rate of change in the number of recorded symptoms, a linear mixed-effects (LME) model with a random participant effect was fit with time as a categorical variable to assess changes at each follow-up time point compared to onset. As a sensitivity analysis, the model above was repeated using the maximum symptom count within each interval to assess robustness to different summarization strategies. All patients with at least one follow-up were included in analyses as mixed effects models are generally robust for unbalanced data across study time points (18). Model assumptions were assessed using diagnostic plots, including residual-vs.-fitted and Q-Q plots using R package “DHARMa” (19). As sensitivity analyses, Poisson and negative binomial mixed-effects models were also fit. No formal adjustments for multiple comparisons were applied as analyses were exploratory in nature. We defined statistical significance using a two-sided *p*-values <0.05. All statistical analyses were conducted in R Studio 4.5.0.

## Results

### Demographics and baseline characteristics

During the study period, 123 children met eligibility criteria and all of them were included in the analysis. Alternative diagnoses which would have constituted exclusion criteria, were not observed among those presenting to the clinic. Mean age is 13.1 years (SD 3.9); 63 patients (51%) were males. Data about race and ethnicity were limited in our study (Table 1). Symptoms developed, on average, 5.1 weeks (SD 13.8) following SARS-CoV-2 infection. At the time of presentation to the clinic, 67 patients (56%) had experienced long COVID symptoms for 0–24 weeks, 34 patients (28%) for 25–52 weeks, and 19 patients (16%) for more than 52 weeks. The exact duration of symptoms at presentation was unknown for 3 patients.

**Table 1:** Demographic and vaccination data among the study population.

Characteristic	Total, *N* = 123
Age in years; mean (SD)	13.1 (3.9)
Sex; *n* (%)	
Male	63 (51%)
Female	60 (49%)
Race; *n* (%)	
African American	2 (6%)
White	16 (47%)
Others/mixed	16 (47%)
*Missing*	89
Ethnicity; *n* (%)	
Hispanic/Latino	15 (39%)
*Missing*	85
Onset of long COVID after SARS-CoV-2 infection (weeks); mean (SD)	5.1 (13.8)
*Missing*	7
Duration of symptoms when first seen in clinic (weeks); *n* (%)	
0–24 weeks	67 (56%)
25–52 weeks	34 (28%)
>52 weeks	19 (16%)
*Missing*	3
Vaccination status of the cohort (*N* = 123); *n* (%; 95% CI)	
Vaccinated	82 (80%; 70%–87%)
Unvaccinated	21 (20%; 13%–30%)
*Missing*	20
Timing of vaccination in relation to the onset of long COVID (*N* = 82); *n* (%; 95% CI)	
Vaccinated after onset of long COVID	26 (33%; 23%–45%)
Vaccinated before onset of long COVID	53 (67%; 55%–77%)
*Missing*	3
Course of symptoms after vaccination (*N* = 26)	
No change in symptoms	10 (59%; 33%–81%)
Improvement in symptoms	7 (41%; 19%–67%)
*Missing*	9

### Symptom prevalence at presentation

The most commonly reported symptoms were fatigue (93%), headache (70%), exercise intolerance (53%), dizziness (44%), brain fog (41%), musculoskeletal pain (29%), shortness of breath and abdominal pain (28%), palpitations (26%), and chest pain and nausea (23%) (Table 2). Fatigue was the most frequently reported and persistent symptom, with prevalence rates of 33% at 6 months (*N* = 24), 42% between 6 and 12 months (*N* = 33), and 62% between 12 and 24 months (*N* = 26) after symptom onset.

**Table 2:** Clinical characteristics of long COVID and sex differences among the study population.

Symptom	Overall *N* = 123^a^	Female *N* = 60^a^	Male *N* = 63^a^	*p*-value^b^	OR (95% CI) (ref – Male)
Fatigue	114 (93%)	56 (93%)	58 (92%)	>0.9	1.2 (0.3, 4.6)
Headache	86 (70%)	45 (75%)	41 (65%)	0.2	1.6 (0.7, 3.5)
Exercise intolerance	65 (53%)	37 (62%)	28 (44%)	0.071	2.0 (1.0, 4.1)
Dizziness	54 (44%)	36 (60%)	18 (29%)	<0.001	3.7 (1.8, 7.8)
Brain fog	50 (41%)	30 (50%)	20 (32%)	0.045	2.1 (1.0, 4.4)
Musculoskeletal pain	36 (29%)	22 (37%)	14 (22%)	0.11	2.0 (0.9, 4.4)
Shortness of breath	35 (28%)	22 (37%)	13 (21%)	0.071	2.2 (1.0, 4.9)
Abdominal pain	35 (28%)	20 (33%)	15 (24%)	0.3	1.6 (0.7, 3.5)
Palpitations	32 (26%)	24 (40%)	8 (13%)	<0.001	4.4 (1.9, 11.1)
Chest pain	28 (23%)	18 (30%)	10 (16%)	0.085	2.2 (1.0, 5.4)
Nausea	28 (23%)	18 (30%)	10 (16%)	0.085	2.2 (1.0, 5.4)
Insomnia	24 (20%)	12 (20%)	12 (19%)	>0.9	1.1 (0.4, 2.6)
Anxiety^c^	23 (19%)	12 (20%)	11 (18%)	0.8	1.2 (0.5, 2.9)
Weight loss^c^	20 (17%)	8 (14%)	12 (19%)	0.5	0.7 (0.2, 1.7)
Loss of appetite	19 (15%)	4 (6.7%)	15 (24%)	0.012	0.2 (0.1, 0.7)
Blurry vision	17 (14%)	11 (18%)	6 (9.5%)	0.2	2.1 (0.7, 6.1)
Anosmia	17 (14%)	9 (15%)	8 (13%)	0.8	1.2 (0.4, 3.3)
Hypersomnia	17 (14%)	8 (13%)	9 (14%)	>0.9	0.9 (0.3, 2.5)
Persistent fever	15 (12%)	7 (12%)	8 (13%)	>0.9	0.9 (0.3, 2.6)
Vomiting	14 (11%)	6 (10%)	8 (13%)	0.8	0.8 (0.3, 2.3)
Cough	13 (11%)	6 (10%)	7 (11%)	>0.9	0.9 (0.3, 2.8)
Sore throat	12 (9.8%)	4 (6.7%)	8 (13%)	0.4	0.5 (0.1, 1.7)
Altered taste	12 (9.8%)	7 (12%)	5 (7.9%)	0.6	1.5 (0.5, 5.0)
Rash	12 (9.8%)	5 (8.3%)	7 (11%)	0.8	0.7 (0.2, 2.4)
Loss of taste^c^	11 (9.0%)	4 (6.7%)	7 (11%)	0.5	0.6 (0.2, 2.0)
Passing out	11 (8.9%)	9 (15%)	2 (3.2%)	0.027	4.5 (1.2, 24.5)
Low mood	11 (8.9%)	6 (10%)	5 (7.9%)	0.8	1.3 (0.4, 4.4)
POTS	10 (8.1%)	8 (13%)	2 (3.2%)	0.050	4.0 (1.0, 21.8)

^a^ *n* (%).

^b^ Fisher's exact test.

^c^ 1–2 participants with missing data; missing data is excluded from the denominator.

Patients were stratified into two age groups: a younger group (<12 years; *n* = 36, 29%) and an adolescent group (>12 years; *n* = 87, 71%). Fatigue and headache remained the most frequently reported symptoms across both groups, with no statistically significant difference observed between males and females. Fatigue was reported by 81% of the younger group and 98% of the adolescent group, while headaches were reported by 58% of the younger children and 75% of the adolescents.

### Sex differences in symptoms

There was no difference between males and females in symptom duration at presentation (*p* = 0.8). However, females were significantly more likely to experience dizziness [*p* < 0.001; OR (95% CI): 3.7 (1.8, 7.8)], brain fog [*p* = 0.04; OR (95% CI): 2.1 (1.0, 4.4)], palpitations [*p* < 0.001; OR (95% CI): 4.4 (1.9, 11.1)], and a diagnosis of postural orthostatic tachycardia syndrome (POTS) [*p* = 0.05; OR (95% CI): 4.0 (1.0, 21.8)]. In contrast, females were significantly less likely to report loss of appetite [*p* = 0.012; OR (95% CI): 0.2 (0.1, 0.7)] (Table 2). Upon stratification by age, chest pain was significantly more prevalent among females in the younger group [50% vs. 4.5%, *p* = 0.003; OR (95% CI): 14.3 (2.5, 153.5)] (Table 3). In the adolescent group, females reported higher frequencies of dizziness [65% vs. 34%, *p* = 0.005; OR (95% CI): 3.5 (1.5, 8.6)] and palpitations [43% vs. 15%, *p* = 0.005; OR (95% CI): 4.2 (1.6, 12.5)] compared to males. In contrast, females were significantly less likely to report loss of appetite [*p* = 0.01; OR (95% CI): 0.2 (0.0, 0.6)] (Table 4).

**Table 3:** Clinical characteristics of long COVID and sex differences among patients under 12 years of age.

Symptom	Overall *N* = 36^a^	Female *N* = 14^a^	Male *N* = 22^a^	*p*-value^b^	OR (95% CI) (ref – Male)
Fatigue	29 (81%)	11 (79%)	18 (82%)	>0.9	0.8 (0.2, 4.2)
Headache	21 (58%)	10 (71%)	11 (50%)	0.3	2.3 (0.6, 9.9)
Exercise intolerance	19 (53%)	9 (64%)	10 (45%)	0.3	2.1 (0.6, 8.2)
Dizziness	10 (28%)	6 (43%)	4 (18%)	0.14	3.1 (0.8, 14.3)
Brain fog	11 (31%)	7 (50%)	4 (18%)	0.067	4.1 (1.0, 18.8)
Musculoskeletal pain	10 (28%)	6 (43%)	4 (18%)	0.14	3.1 (0.8, 14.3)
Shortness of breath	9 (25%)	6 (43%)	3 (14%)	0.11	4.3 (1.0, 21.9)
Abdominal pain	8 (22%)	4 (29%)	4 (18%)	0.7	1.8 (0.4, 8.3)
Palpitations	6 (17%)	4 (29%)	2 (9.1%)	0.2	3.5 (0.7, 23.1)
Chest pain	8 (22%)	7 (50%)	1 (4.5%)	0.003	14.3 (2.5, 153.5)
Nausea	–	–	–	–	–
Insomnia	–	–	–	–	–
Anxiety^c^	8 (23%)	4 (31%)	4 (18%)	0.4	1.9 (0.4, 9.3)
Weight loss^c^	–	–	–	–	–
Loss of appetite	7 (19%)	2 (14%)	5 (23%)	0.7	0.6 (0.1, 3.2)
Blurry vision	–	–	–	–	–
Anosmia	–	–	–	–	–
Hypersomnia	–	–	–	–	–
Persistent fever	–	–	–	–	–
Vomiting	–	–	–	–	–
Cough	7 (19%)	2 (14%)	5 (23%)	0.7	0.6 (0.1, 3.2)
Sore throat	–	–	–	–	–
Altered taste	–	–	–	–	–
Rash	–	–	–	–	–
Loss of taste	–	–	–	–	–
Passing out	–	–	–	–	–
Low mood	–	–	–	–	–
POTS	–	–	–	–	–

^a^ *n* (%).

^b^ Fisher's exact test.

^c^ 1 participant with missing data; missing data is excluded from the denominator.

**Table 4:** Clinical characteristics of long COVID and sex differences among patients 12 years of age or older.

Symptom	Overall *N* = 87^a^	Female *N* = 46^a^	Male *N* = 41^a^	*p*-value^b^	OR (95% CI) (ref – Male)
Fatigue	85 (98%)	45 (98%)	40 (98%)	>0.9	1.1 (0.1, 14.2)
Headache	65 (75%)	35 (76%)	30 (73%)	0.8	1.2 (0.4, 3)
Exercise intolerance	46 (53%)	28 (61%)	18 (44%)	0.14	2.0 (0.8, 4.6)
Dizziness	44 (51%)	30 (65%)	14 (34%)	0.005	3.5 (1.5, 8.6)
Brain fog	39 (45%)	23 (50%)	16 (39%)	0.4	1.5 (0.7, 3.6)
Musculoskeletal pain	26 (30%)	16 (35%)	10 (24%)	0.4	1.6 (0.7, 4.2)
Shortness of breath	26 (30%)	16 (35%)	10 (24%)	0.4	1.6 (0.7, 4.2)
Abdominal pain	27 (31%)	16 (35%)	11 (27%)	0.5	1.4 (0.6, 3.6)
Palpitations	26 (30%)	20 (43%)	6 (15%)	0.005	4.2 (1.6, 12.5)
Chest pain	20 (23%)	11 (24%)	9 (22%)	>0.9	1.1 (0.4, 3.0)
Nausea	23 (26%)	16 (35%)	7 (17%)	0.088	2.5 (0.9, 7.0)
Insomnia	19 (22%)	9 (20%)	10 (24%)	0.6	0.8 (0.3, 2.1)
Anxiety	15 (17%)	8 (17%)	7 (18%)	>0.9	1.0 (0.3, 3.0)
Weight loss	16 (18%)	7 (15%)	9 (22%)	0.6	0.6 (0.2, 1.9)
Loss of appetite	12 (14%)	2 (4.3%)	10 (24%)	0.011	0.2 (0.0, 0.6)
Blurry vision	12 (14%)	7 (15%)	5 (12%)	0.8	1.3 (0.4, 4.4)
Anosmia	13 (15%)	6 (13%)	7 (17%)	0.8	0.7 (0.2, 2.3)
Hypersomnia	13 (15%)	8 (17%)	5 (12%)	0.6	1.5 (0.5, 5.0)
Persistent fever	10 (11%)	5 (11%)	5 (12%)	>0.9	0.9 (0.2, 3.2)
Vomiting	10 (11%)	5 (11%)	5 (12%)	>0.9	0.9 (0.2, 3.2)
Cough	6 (6.9%)	4 (8.7%)	2 (4.9%)	0.7	1.7 (0.3, 10.0)
Sore throat	8 (9.2%)	2 (4.3%)	6 (15%)	0.14	0.3 (0.1, 1.3)
Altered taste	10 (11%)	5 (11%)	5 (12%)	>0.9	0.9 (0.2, 3.2)
Rash	8 (9.2%)	4 (8.7%)	4 (9.8%)	>0.9	0.9 (0.2, 3.6)
Loss of taste^c^	9 (10%)	3 (6.5%)	6 (15%)	0.3	0.4 (0.1, 1.6)
Passing out	9 (10%)	7 (15%)	2 (4.9%)	0.2	3.0 (0.7, 16.8)
Low mood	8 (9.2%)	3 (6.5%)	5 (12%)	0.5	0.5 (0.1, 2.1)
POTS	7 (8.0%)	6 (13%)	1 (2.4%)	0.11	4.3 (0.9, 42.9)

^a^ *n* (%).

^b^ Fisher's exact test.

^c^ 1 participant with missing data; missing data is excluded from the denominator.

### Longitudinal symptom trajectories

**Figure 1:**
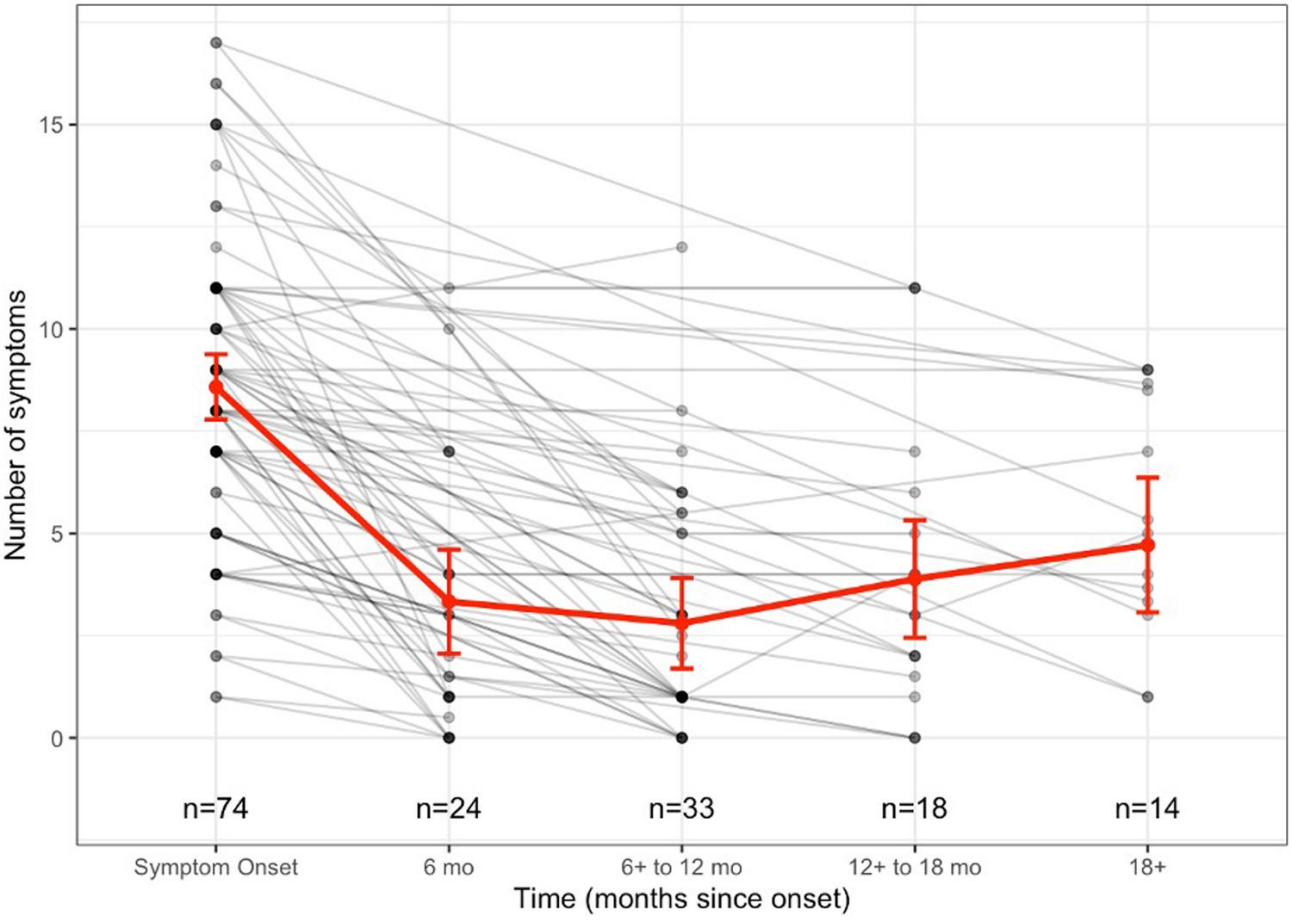
Individual symptom trajectories over time with model-based mean estimates. Each gray line represents an individual participant's number of reported symptoms at each follow-up interval. Overlapping points occur when multiple participants reported the same number of symptoms at a given timepoint. The red line with error bars represents the estimated mean symptom trajectory from the linear mixed-effects model, with 95% confidence intervals.

**Figure 2:**
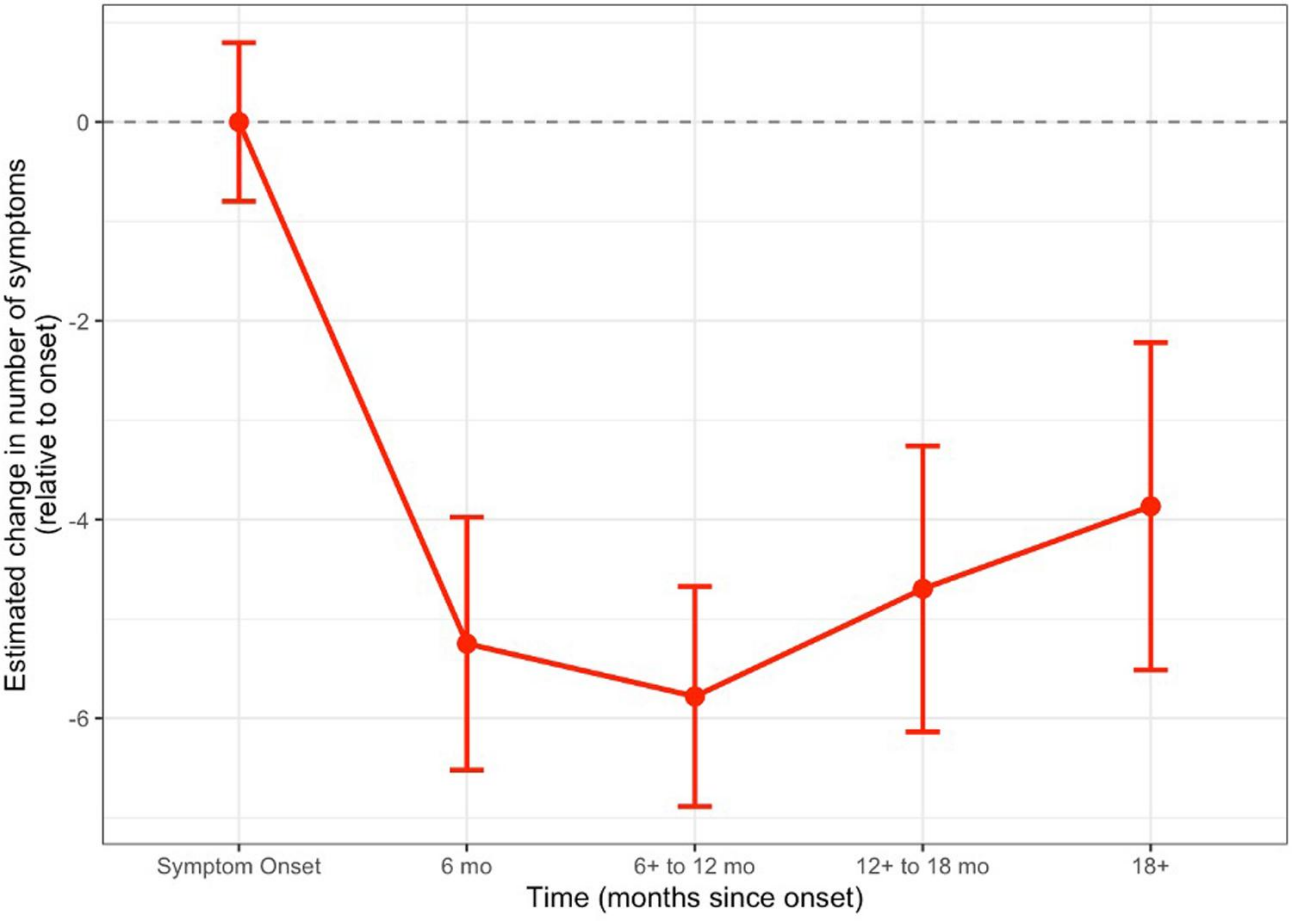
Model-based estimates of change in number of symptoms over time since onset. The red line with error bars represents the estimated mean change in symptom count from onset at key time intervals from the linear mixed-effects model, with 95% confidence intervals.

Of the 123 patients, 74 patients had at least one follow-up clinic visit; the overall trend in reported symptoms over time is shown in Figure 1. No significant differences in demographics and characteristics were found between patients with and without follow-up (Supplementary Table S1). The residuals from the primary linear mixed-effects (LME) model were approximately normally distributed, with no substantial deviations from homoscedasticity or linearity observed (Supplementary Figure S1). As sensitivity analyses, Poisson and negative binomial mixed-effects models were also fit. The Poisson model exhibited significant deviations from uniformity and dispersion, indicating poor fit. In contrast, the negative binomial mixed-effects model showed residual patterns similar to the LME model, with no evidence of significant deviation from assumptions. Results were consistent across the LME and negative binomial approaches. Given comparable fit, the LME model was retained as the primary analytic model. Among the 74 patients, the estimated mean number of symptoms at the onset of long COVID from the LME model was 8.6 (95% CI: 7.8–9.4). When compared to symptom onset, the mean number of symptoms decreased by 5.3 (95% CI: −6.5 to −4.0) at 6 months, by 5.8 (95% CI: −6.9 to −4.7) at 6–12 months, by 4.7 (95% CI: −6.1 to −3.3) at 12–18 months, and by 3.9 (95% CI: −5.4 to −2.2) at 18 months and beyond (*p* < 0.001) (Figure 2). Results from the sensitivity analysis using the maximum symptom count were largely consistent with the primary analysis. Estimates differed by only one decimal place across intervals, except for the 18+ month interval (3.9 vs. 2.9), supporting the robustness of the primary findings across different symptom summary methods.

### Symptom domains

Fatigue/exercise intolerance (94%) and neurologic symptoms (77%) were the most frequently reported domains (Supplementary Table S2). Cardiovascular symptoms were significantly more common in females than males [68% vs. 30%, *p* < 0.001; OR (95% CI): 4.9 (2.3, 10.6)]. Other domains—including musculoskeletal, gastrointestinal, respiratory, sleep disturbance, ENT, psychological, and dermatologic symptoms—did not differ significantly by sex. When stratified by age, patterns were similar. In children <12 years, fatigue (83%) and neurologic symptoms (72%) predominated, with no significant sex differences. Among those ≥12 years, fatigue (97%) and neurologic symptoms (80%) remained most common, and cardiovascular symptoms continued to show a strong female predominance [*p* < 0.001; OR (95% CI): 4.7 (2.0, 12.0)].

### COVID-19 vaccination and long COVID

Of the 123 patients included in the study, 82 (80%) had received at least the primary COVID-19 vaccine series, 21 (20%) were unvaccinated, and vaccination status was unknown for 20 patients (Table 1). Among those who completed the primary vaccine series, 31 patients (38%) had not received any booster doses, while 51 (62%) had received at least one booster. There was no association between COVID-19 vaccination status and long COVID symptom duration (*p* = 0.4) on their initial presentation to the clinic as shown in Supplementary Table S3.

Among the 82 vaccinated patients in our study, 53 (67%) received the COVID-19 vaccine before the onset of long COVID symptoms and 26 (33%) developed long COVID symptoms prior to receiving the COVID-19 vaccine (Table 1). Of the 26 patients who received the COVID-19 vaccine after the onset of long COVID symptoms, 7 (41%) reported subjective improvement at subsequent follow-up visits, 10 (59%) reported no change in their symptoms at the time of reassessment, and 9 had no documentation regarding symptom progression in their medical records (Table 1).

Some preliminary findings from this study have been previously presented in abstract form at Open Forum Infectious Diseases (20). It was based on preliminary data that included a smaller subset of patients (with data only through April 2023). The current manuscript expands upon that preliminary report by including a larger sample size, extended data collection through November 2023, detailed analyses of follow-up visits, and comprehensive vaccination data.

## Discussion

Long COVID is now increasingly recognized as a significant long-term health concern in the pediatric population. In this study, we describe the clinical characteristics of children with long COVID, based on a substantial number of patients evaluated at a dedicated long COVID clinic. Adolescents represented the majority of affected individuals, with fatigue and headache identified as the most prevalent symptoms.

Symptom profiles in our study are comparable to prior reports published on pediatric long COVID. A nationwide cohort study by Borch et al. (1) showed that between 4% and 66% of children experienced lingering symptoms following acute COVID-19. Reported symptoms included fatigue, sleep disturbances, respiratory issues, nasal congestion, musculoskeletal pain, cognitive difficulties, and changes in smell or taste (1).

A systematic review conducted by Jiang et al. (21) noted over 20 persistent symptoms and clinical features in children and adolescents following SARS-CoV-2 infection. Approximately 16.2% of the pediatric population experienced at least one lingering symptom three months after infection (21).

A meta-analysis conducted by Behnood et al. (11) examined the prevalence and duration of long COVID symptoms in a cohort of 23,141 children and adolescents and found that fatigue (47%), shortness of breath (43%), and headache (35%) emerged as the most commonly reported symptoms. Furthermore, when compared to control groups, the incidence of cognitive difficulties, headache, anosmia, sore throat, and sore eyes was significantly higher in those with a history of COVID-19 (5, 11). A large national case-control study from Denmark reported that children with a history of SARS-CoV-2 infection were more likely than controls to experience at least one symptom persisting beyond two months. While this association was statistically significant across all age groups, the differences in overall somatic symptom burden were small and not considered clinically relevant (22). Our study did not include a control group for comparative analysis.

Sex based differences in the severity of symptoms have been seen in adults with long COVID but not reported in children. In the NIH RECOVER-Adult prospective cohort study, female sex was linked to a higher risk of developing long COVID compared to males (23). Also, a study by Onieva et al. (24) found that females exhibited a higher risk of persistent symptoms and displayed distinct patterns in symptom clusters and functional status compared to males We did not find differences in the duration of symptoms; however, females were more likely to report symptoms like brain fog, dizziness, palpitations, and receive a diagnosis of POTS. A systematic review conducted by Jiang et al. (21) also suggested a potential association between female sex and an increased likelihood of developing certain long COVID symptoms (21).

The LongCOVIDKidsDK study is a national cross-sectional investigation of SARS-CoV-2-positive children aged 0–14 years and matched controls in Denmark had interesting findings. No significant sex-based differences in the prevalence of persistent symptoms were observed among children aged 0–3 and 4–11 years. However, in the 12–14-year age group, both cases and controls showed that girls reported more symptoms than boys. Adolescent girls are generally known to report a greater burden of symptoms and health-related complaints compared to boys, a pattern that typically emerges during puberty and may be influenced by hormonal factors (22). These findings are consistent with our study, which demonstrated a higher prevalence of symptoms among adolescent females.

Long-term follow-up studies in children are limited; few that had follow-up found that most symptoms improved after 6–8 months; however, fatigue and decline in school performance persisted (25). Few pediatric studies have examined outcomes beyond 12 months (26).

Our study addressed the trend of symptoms for 74 patients who had at least one follow-up visit up to ≥18 months. We noted a trend for symptom reduction over time in this patient population, however, there was a subset that continues to struggle with persistent symptoms. These findings are in line with a prior study that included 1,243 children and found that 11.5% remained symptomatic at six months, 3% at 12 months, and 1.2% at 18 months (27).

The relationship between COVID-19 vaccination and long COVID in children is an emerging research area. Our study provided descriptive data on long COVID symptomatology and the potential impact of COVID-19 vaccination. However, given the observational nature of the design, definitive conclusions and clinical recommendations could not be established. At initial clinic presentation, no association was found between COVID-19 vaccination status and long COVID symptom duration (*p* = 0.4). This may be attributed to variations in the timing of patient presentation, differences in vaccination schedules, and the number of booster doses received.

A systematic review by Gao et al. (16) explored the relationship between vaccination and long COVID. The study showed that the COVID-19 vaccines had an effect of reducing the risk of long COVID in individuals vaccinated either before or after SARS-CoV-2 infection. The effect was seen in participants vaccinated with two doses, but not one dose. Of note, the patient population included in this systematic review included both adults and children (16). In a large retrospective study, Razzaghi et al. (28) evaluated the effectiveness of COVID-19 vaccination in preventing long COVID among children, indicating a moderate protective benefit, particularly in adolescents, who are at greater risk for developing long COVID. However, the protective effect appeared to diminish over time (28).

Another study by Yousaf et al. (29) in Children Aged 5–17 Years found that COVID-19 vaccination was associated with significantly reduced odds of developing post-COVID condition (PCC) symptoms in children. Adjusted odds ratios indicated a 57% reduction in the likelihood of having at least one PCC symptom and a 73% reduction for two or more symptoms among vaccinated compared to unvaccinated children.

These results highlight potential additional benefits of vaccination that extend beyond protection from acute illness and may support broader pediatric vaccine uptake. When we analyzed the potential impact of the COVID-19 vaccine on children who received the vaccine after the onset of long COVID, we found that almost 41% reported symptom improvement over the few weeks following vaccination. While this is an interesting observation, it must be noted that data is subjective and based on patient and caregiver responses on follow-up in clinic, and not based on a validated questionnaire, and our study was not designed to study the vaccine impact.

Some studies in adults have also evaluated the impact of the vaccine on long COVID symptoms, and a meta-analysis reported that 20% of the adults with long COVID who received the COVID-19 vaccine reported improvement post-vaccine (30). Our study population is too small to conclude that there are notable differences in response to the vaccine in children compared to adults, but this is an important question that should be addressed in larger, prospective studies.

Our study has several limitations. It is a single-center study, predominantly involving self-referred patients, which may lead to an overrepresentation of more severely affected individuals or those with better access to healthcare. Additionally, our study is constrained by limited epidemiological data, which precluded meaningful analysis of potential associations with long COVID outcomes and prevented us from assessing whether long COVID outcomes vary across demographic groups. We also recognize that differences in follow-up rates may reflect selection bias and residual confounding effects from uncollected socio-economic factors that were unavailable in our chart review. The absence of a control group limits our ability to draw comparative conclusions. The lack of validated questionnaires for symptom assessment and quality of life evaluation is another limitation. Also, the absence of standardized tools for symptom severity measurement hindered us from conducting a quantitative analysis of symptom burden and comparing outcomes across patients. Furthermore, the assessment of “symptom improvement” following post- vaccination relied on retrospective chart documentation from only 26 children. As such, these findings are subject to both recall bias and reporting bias, since they depend on caregiver and clinician interpretation rather than standardized symptom assessment so no firm conclusions regarding vaccine impact can be drawn from this data. Future research should focus on more structured, well-designed studies with prospective follow-up on larger sample sizes to validate our findings. To better understand the effect of vaccines on long COVID symptoms, a systematic assessment that accounts for confounding factors and other interventions will be necessary to accurately determine the vaccine's contribution.

While our study has limitations, strengths include a uniform approach to the diagnosis as it is a single-site, single-clinic study, longitudinal follow-up data in patients who presented for follow-up, and long COVID diagnosis made by physicians after considering and eliminating other potential reasons for presentation. Many of the large multisite electronic health record-based studies that rely on a computable phenotype may capture patients with long COVID-like symptoms and thus may be more widely inclusive of other conditions, and therefore likely less reliable.

While our study is confined to a single center, its findings emphasize the critical need for continued research into the symptomatology of pediatric long COVID. Understanding the potential therapeutic impact of vaccines is particularly important as we seek to identify effective interventions. Comprehensive studies will be essential to inform the development of targeted support strategies, ensuring that children affected by this condition receive the care and resources they need to manage and recover from long COVID.

## Conclusion

This study highlights the broad clinical spectrum of long COVID in children and adolescents, with fatigue and headache emerging as the most common and persistent symptoms across all age groups. While many patients experienced gradual symptom improvement over time, a subset continued to report significant impairment well beyond the acute illness. No clear association was observed between COVID-19 vaccination status and symptom duration at initial presentation, though the potential for symptom improvement following vaccination warrants further investigation. The observed age and gender differences in symptom profiles underscore the importance of individualized assessment and multidisciplinary care. Overall, these findings emphasize the need for continued research and long-term follow-up to better understand the natural history of pediatric long COVID and to inform effective management strategies.

## Data Availability

The raw data supporting the conclusions of this article will be made available by the authors, without undue reservation.
