# Supplementary material for: Exploring long COVID in pediatric patients: clinical insights from a long COVID clinic
Source: Front Pediatr. 2025 Oct 15;13:1640747. doi: 10.3389/fped.2025.1640747 (PMC12568432; doi:10.3389/fped.2025.1640747)

**Supplemental Table 1.** Comparison of Demographics and Vaccination Status Between Patients With and Without Follow-Up

| **Characteristic** | **Total, N = 123** | **At least 1 follow-up, N = 74** | **0 follow-up, N = 49** | **p-value*^1^*** |
| --- | --- | --- | --- | --- |
| **Age in years; mean (SD)** | 13.1 (3.9) | 13.3 (3.5) | 12.9 (4.5) | 0.9 |
| **Sex; n (%)** |  |  |  | 0.5 |
| Male | 63 (51%) | 36 (49%) | 27 (55%) |  |
| Female | 60 (49%) | 38 (51%) | 22 (45%) |  |
| **Race; n (%)** |  |  |  | 0.3 |
| African American | 2 (6%) | 2 (10%) | 0 (0%) |  |
| White | 16 (47%) | 7 (35%) | 9 (64%) |  |
| Others/mixed | 16 (47%) | 11 (55%) | 5 (36%) |  |
| *Missing* | 89 | 54 | 35 |  |
| **Ethnicity; n (%)** |  |  |  | 0.7 |
| Hispanic/Latino | 15 (39%) | 10 (42%) | 5 (36%) |  |
| *Missing* | 85 | 50 | 35 |  |
| **Onset of long COVID after SARS-CoV-2 infection (weeks); mean (SD)** | 5.1 (13.8) | 3.0 (7.4) | 8.0 (19.5) | 0.3 |
| *Missing* | 7 | 5 | 2 |  |
| **Duration of symptoms when first seen in clinic (weeks); n (%)** |  |  |  | 0.8 |
| 0-24 weeks | 67 (56%) | 41 (55%) | 26 (57%) |  |
| 25-52 weeks | 34 (28%) | 20 (27%) | 14 (30%) |  |
| > 52 weeks | 19 (16%) | 13 (18%) | 6 (13%) |  |
| *Missing* | 3 | 0 | 3 |  |
| **Vaccination status of the cohort**  **(N=123); n (%; 95% CI)** |  |  |  | 0.5 |
| Vaccinated | 82 (80%; 70% - 87%) | 50 (82%; 70% - 90%) | 32 (76%; 60% - 87%) |  |
| Unvaccinated | 21 (20%; 13% - 30%) | 11 (18%; 10% - 30%) | 10 (24%; 13% - 40%) |  |
| *Missing* | 20 | 13 | 7 |  |
| **Timing of vaccination in relation to the onset of long COVID**  **(N=82); n (%; 95% CI)** |  |  |  | 0.2 |
| Vaccinated after onset of long COVID | 26 (33%; 23% - 45%) | 18 (38%; 25% - 54%) | 8 (25%; 12% - 44%) |  |
| Vaccinated before onset of long COVID | 53 (67%; 55% - 77%) | 29 (62%; 46% - 75%) | 24 (75%; 56% - 88%) |  |
| *Missing* | 3 | 3 | 0 |  |
| **Course of symptoms after vaccination**  **(N=26)** |  |  |  | 0.6 |
| No change in symptoms | 10 (59%; 33% - 81%) | 7 (54%; 26% - 80%) | 3 (75%; 22% - 99%) |  |
| Improvement in symptoms | 7 (41%; 19% - 67%) | 6 (46%; 20% - 74%) | 1 (25%; 1% - 78%) |  |
| *Missing* | 9 | 5 | 4 |  |

***^1^***Wilcoxon rank sum test; Fisher’s exact test

**Supplemental Table 2.** Long COVID symptom domains and sex differences among the study population and by age groups.

| **Full Cohort** | | | | | |
| --- | --- | --- | --- | --- | --- |
| **Domain** | **Overall** N = 123*^1^* | **Female** N = 60*^1^* | **Male** N = 63*^1^* | **p-value***^2^* | **OR (95% CI) (ref – Male)** |
| Fatigue/exercise intolerance | 116 (94%) | 57 (95%) | 59 (94%) | >0.9 | 1.2 (0.3, 5.8) |
| Neurologic | 97 (79%) | 49 (82%) | 48 (76%) | 0.5 | 1.4 (0.6, 3.3) |
| Cardiovascular | 60 (49%) | 41 (68%) | 19 (30%) | <0.001 | 4.9 (2.3, 10.6) |
| Musculoskeletal | 55 (45%) | 29 (48%) | 26 (41%) | 0.5 | 1.3 (0.7, 2.7) |
| Gastrointestinal | 50 (41%) | 26 (43%) | 24 (38%) | 0.6 | 1.2 (0.6, 2.5) |
| Respiratory | 50 (41%) | 26 (43%) | 24 (38%) | 0.6 | 1.2 (0.6, 2.5) |
| Constitutional | 43 (35%) | 17 (28%) | 26 (41%) | 0.2 | 0.6 (0.3, 1.2) |
| Sleep disturbance | 40 (33%) | 20 (33%) | 20 (32%) | >0.9 | 1.1 (0.5, 2.3) |
| ENT | 35 (28%) | 15 (25%) | 20 (32%) | 0.4 | 0.7 (0.3, 1.6) |
| Psychological | 25 (20%) | 13 (22%) | 12 (19%) | 0.8 | 1.2 (0.5, 2.8) |
| Eye symptoms | 17 (14%) | 11 (18%) | 6 (9.5%) | 0.2 | 2.1 (0.7, 6.1) |
| Dermatology | 15 (12%) | 6 (10%) | 9 (14%) | 0.6 | 0.7 (0.2, 2.0) |
| **Age < 12** | | | | | |
| **Domain** | **Overall** N = 36*^1^* | **Female** N = 14*^1^* | **Male** N = 22*^1^* | **p-value***^2^* | **OR (95% CI) (ref – Male)** |
| Fatigue/exercise intolerance | 30 (83%) | 11 (79%) | 19 (86%) | 0.7 | 0.6 (0.1, 3.2) |
| Neurologic | 26 (72%) | 12 (86%) | 14 (64%) | 0.3 | 2.9 (0.7, 17.8) |
| Cardiovascular | 11 (31%) | 7 (50%) | 4 (18%) | 0.067 | 4.1 (1.0, 18.8) |
| Musculoskeletal | 17 (47%) | 8 (57%) | 9 (41%) | 0.5 | 1.9 (0.5, 7.2) |
| Gastrointestinal | 13 (36%) | 4 (29%) | 9 (41%) | 0.5 | 0.6 (0.1, 2.4) |
| Respiratory | 14 (39%) | 7 (50%) | 7 (32%) | 0.3 | 2.1 (0.5, 8.1) |
| Constitutional | 14 (39%) | 5 (36%) | 9 (41%) | >0.9 | 0.8 (0.2, 3.1) |
| Sleep disturbance | 9 (25%) | 3 (21%) | 6 (27%) | >0.9 | 0.8 (0.2, 3.4) |
| ENT | 10 (28%) | 5 (36%) | 5 (23%) | 0.5 | 1.8 (0.4, 7.9) |
| Psychological | 8 (22%) | 4 (29%) | 4 (18%) | 0.7 | 1.8 (0.4, 8.3) |
| Eye symptoms | 5 (14%) | 4 (29%) | 1 (4.5%) | 0.064 | 6.1 (1.0, 67.3) |
| Dermatology | 4 (11%) | 1 (7.1%) | 3 (14%) | >0.9 | 0.6 (0.1, 4.3) |
| **Age 12 or above** | | | | | |
| **Domain** | **Overall** N = 87*^1^* | **Female** N = 46*^1^* | **Male** N = 41*^1^* | **p-value***^2^* | **OR (95% CI) (ref – Male)** |
| Fatigue/exercise intolerance***^3^*** | 86 (99%) | 46 (100%) | 40 (98%) | 0.5 | - |
| Neurologic | 71 (82%) | 37 (80%) | 34 (83%) | 0.8 | 0.9 (0.3, 2.5) |
| Cardiovascular | 49 (56%) | 34 (74%) | 15 (37%) | <0.001 | 4.7 (2.0, 12.0) |
| Musculoskeletal | 38 (44%) | 21 (46%) | 17 (41%) | 0.8 | 1.2 (0.5, 2.8) |
| Gastrointestinal | 37 (43%) | 22 (48%) | 15 (37%) | 0.4 | 1.6 (0.7, 3.7) |
| Respiratory | 36 (41%) | 19 (41%) | 17 (41%) | >0.9 | 1.0 (0.4, 2.3) |
| Constitutional | 29 (33%) | 12 (26%) | 17 (41%) | 0.2 | 0.5 (0.2, 1.2) |
| Sleep disturbance | 31 (36%) | 17 (37%) | 14 (34%) | 0.8 | 1.1 (0.5, 2.7) |
| ENT | 25 (29%) | 10 (22%) | 15 (37%) | 0.2 | 0.5 (0.2, 1.2) |
| Psychological | 17 (20%) | 9 (20%) | 8 (20%) | >0.9 | 1.0 (0.4, 2.9) |
| Eye symptoms | 12 (14%) | 7 (15%) | 5 (12%) | 0.8 | 1.3 (0.4, 4.4) |
| Dermatology | 11 (13%) | 5 (11%) | 6 (15%) | 0.7 | 0.7 (0.2, 2.5) |

*^1^* n (%)

*^2^* Fisher’s exact test

*^3^* No estimated OR due to high prevalence rate.

**Supplemental Table 3.** Comparison of Long COVID Symptom Duration on initial presentation by COVID-19 Vaccination Status.

| Long COVID symptom duration; n (%; 95% CI) | Vaccinated (N=82) | Unvaccinated (N=21) | P value |
| --- | --- | --- | --- |
| 0-24 weeks | 46 (56%; 45% - 67%) | 9 (45%; 24% - 68%) | 0.4 |
| 25- 52 weeks | 25 (30%; 21% - 42%) | 6 (30%; 13% - 54%) |  |
| >52 weeks | 11 (14%; 7% - 23%) | 5 (25%; 10% - 49%) |  |
| *Missing* | 0 | 1 |  |

**Supplemental Figure 1. Diagnostic plots evaluating LME model fit through residual analysis. a) Residuals vs. fitted values plot, assessing homoscedasticity and potential non-linearity and b) QQ plot of residuals indicating no significant deviation from normality and boxplots of standardized residuals across predicted value bins with tests for within-group uniformity and homogeneity of variance showing non-significant results.**


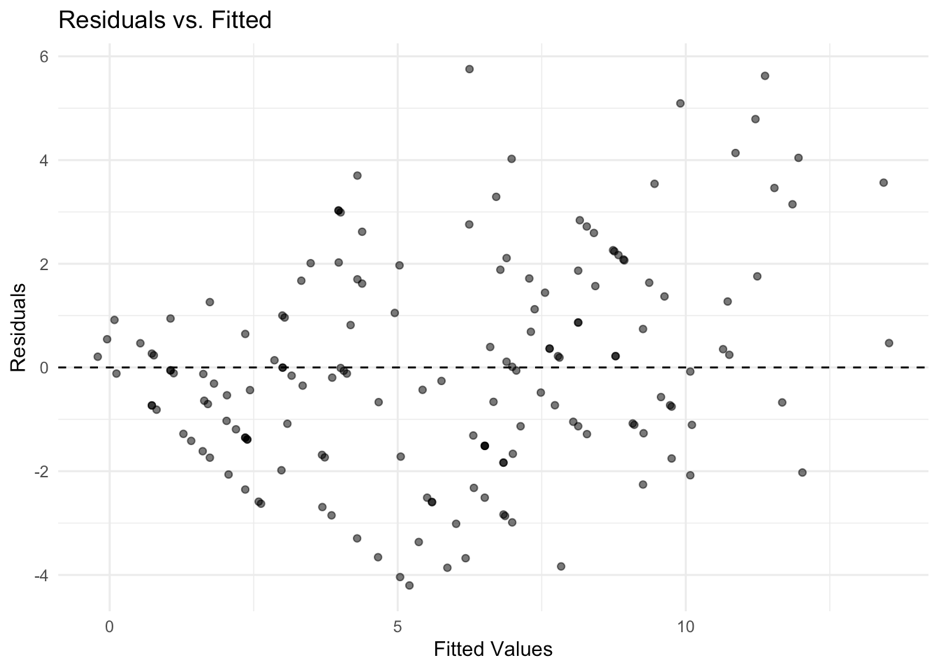


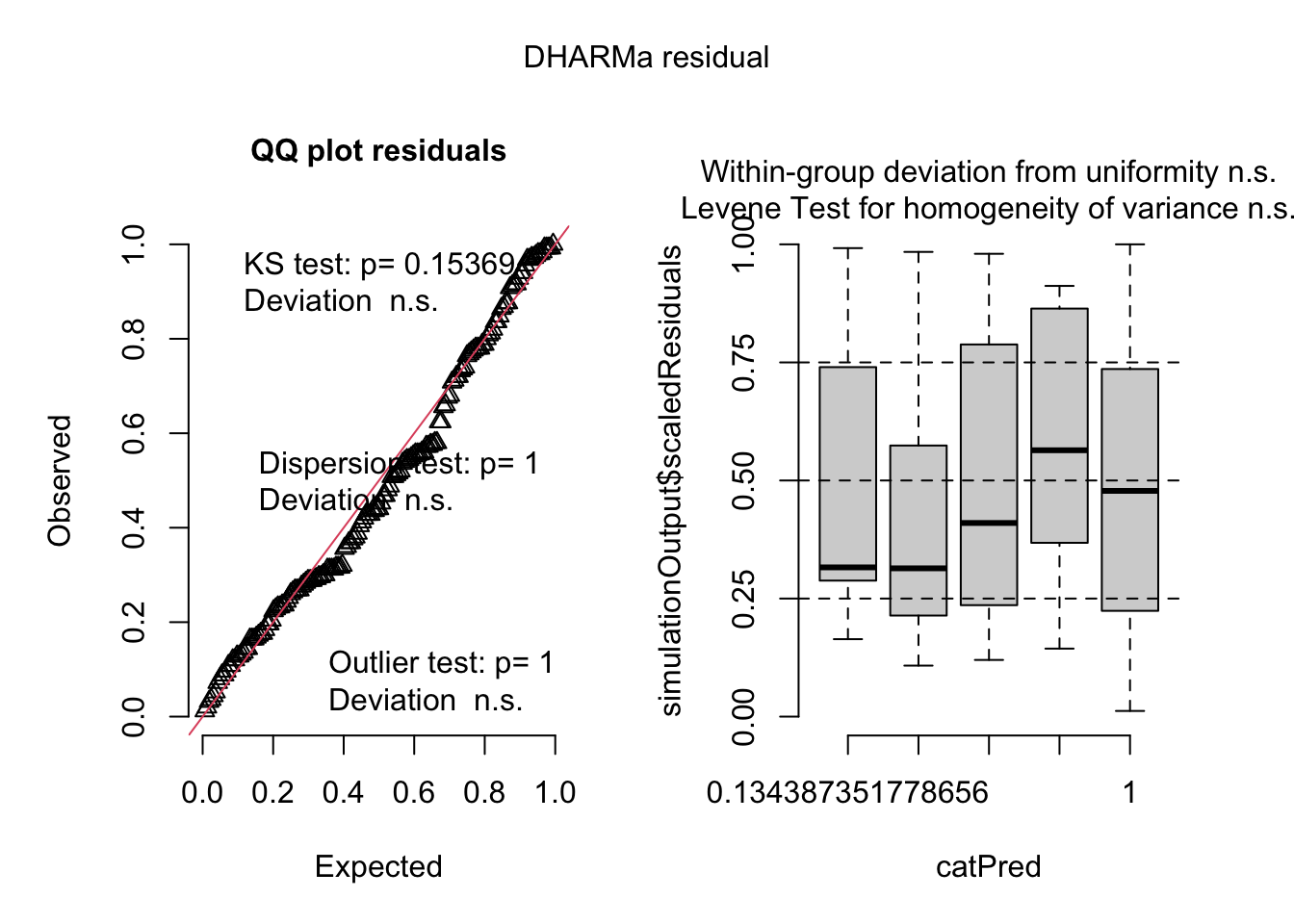

Supplement: Supplementary file 1 [file Table1.docx]
